# Moccasin Bite Treated with Adrenalin Chloride

**Published:** 1905-01

**Authors:** R. Menger

**Affiliations:** San Antonio, Texas


For Texas Medical Journal.

### Moccasin Bite Treated With Adrenalin Chloride.

BY R. MENGER, M. D., SAN ANTONIO, TEXAS.

Last year, in May, in an article of mine in the *Texas Medical Journal* on the rational treatment of inoculated wounds of venomous animals, especially those of the moccasin and rattlesnake, I called attention to the treatment of such wound by injecting a minimal solution of adrenalin chloride near the site of puncture of the poison fangs. This article, though, was based merely on theoretical principles—not having had occasion to try the remedy on a freshly-poisoned case. Shortly, though, after my article was published a general and interesting report on snakebites was published in the above journal by some other colleague, who had tried this adrenalin treatment—as this doctor stated: "According to Menger's suggestion in the *Texas Medical Journal,*" giving a clear history of his case and the decided favorable action of the new drug—the patient recovering rapidly. I am sorry I have not the name of the doctor, as the journal wherein his case was related is not at my disposal just now.

Lately I had occasion to try the new remedy myself on a young Mexican man seriously bitten by a water moccasin. He was bitten in dorsum of right foot, which was very much swollen—due in part also to a tight ligature, and two fang wounds could be seen clearly in lower third of the dorsal part of the foot. The man was brought to the office with his foot tightly ligated around the foot joint, and he was bitten two hours before arriving. When I treated him, in presence of Dr. R. L. Dinwiddie, he seemed to be suffering considerably from the fang wounds, especially on pressure along the wounded surface; he was very pale and pulse hardly perceptible on the other hand, and of slow rate. I injected at once about twenty-five drops of permanganate of potassium solution (2 grains to ℥i, 4 grains to ℥i) inside the fang wounds and, after waiting a while, injected about twenty drops of adrenalin solution (1/1000 solution) near the fang wounds. This was done, of course, whilst the foot joint was still ligated—after also incising the fang wounds to relieve some of the serum and blood. The adrenalin injections I made quite deep into the subcellular tissues, and, after still waiting a while, a piece of absorbent cotton saturated with solution of permanganate potassium was applied freely over the entire dorsum of the foot, and the ligature now removed. The pulse rate at once improved and the man was sent home by his father on a cart. About two hours later I visited him at his home—close to the romantic river of San Antonio, where this accident happened—and I found his condition favorable, with pulse greatly improved, full and beating, and complaining but little over his foot, and there was much less swelling of the foot. After another application of adrenalin (ten drops) subcutaneously, and also later one-half grain morphia to secure night rest, I visited him next morning and found him still more improving, with a little less swelling, full and bouncing pulse, but not abnormally so, and no anesthesia of the foot or toes. In the afternoon I saw him again in company with Dr. Dinwiddie, who also was surprised to find him in such improved condition—without any swelling extending upward, as is usually the case in similar cases treated without adrenalin.

Snake venom inoculation will, of course, depend on certain circumstances: the length of time elapsed since inoculation occurred; whether a ligature had been properly and immediately applied (as in above case, as I was told), and whether the fangs had really penetrated deep enough through the skin to produce venom intoxication to serious extent. The virus of venomous animals principally acts direct or secondarily upon the nerve and heart centers as a great depressant. Stimulating remedies, therefore, such as adrenalin, strychnine, nitroglycerine, alcohol, etc., stimulate the nerve and heart centers; but adrenalin, from clinical, etc., reports, is not alone a powerful vaso-motor constrictor, but also a heart stimulant, and, after injecting near the fang wounds, it retards the circulation in the neighborhood of tissues surrounding the fang wounds, and thereby lessens the absorption of the reptile's venom into the general circulation; provided, of course, that it be used in time and before the ligature is removed.

The physiological action of adrenalin and strychnine, therefore, is such as to retard the paralyzing effect of snake venom and allow the secretory organs of the system to eliminate the poison gradually through the urine and sweat glands, and diuretics and large quantities of saline clysmatus are essential for the latter purpose; also pilocarpine hypodermically. The snake's poison fang through its communication with and contraction of its muscular poison-bladder during the act of striking injects the poison as quickly and readily as the action of a hypodermic syringe, and in such cases where blood vessels were perforated or directly injected, death generally results from the paralyzing heart venom, and it shows the necessity of quick action in applying a tight ligature, etc.

In a case of death from a moccasin bite, some years ago, a young, robust man was bitten in the hand and between the fingers by a genuine water moccasin whilst bathing in a river. The man was brought in a semi-collapsed condition to the city hospital. His fingers, hand and arm were terribly swollen—up to the shoulder joint; gangrenous sloughing, with exposure of the tendons and bones setting in rapidly. Adrenalin then was not thought of, but strychnine and other remedies had no effect, but the effect of the venom was noticed whenever the tight bandage around his arm was loosened, as the pulse would rapidly change and the system brought in a convulsive condition, so that he had to be held down and chloroformed.

Further observation will, of course, be required to note the physiological action of adrenalin in snake bites, and it surely is advisable that especially country practitioners carry a sample of adrenalin for ready use in their armamentarium, and make careful notes and publish same after using the remedy. Seemingly, I used rather a large dose—twenty min.—of the solution, but I believe even larger doses are tolerated, on account of the depressed conditions in a severe and genuine snake bite, at least for the first injection, and it can be controlled later on, according to symptoms, etc., presenting themselves.

Of course, other appliances, such as immersing the wounded parts in a warm solution of permanganate of potassium or chlor. sod. i, hot poultices (which were used in above case, also) diuretics, etc., should not be neglected when conditions call for same.

In late years snake bites and deaths from same have considerably lessened with the culture and upbuilding of the vast area of Texas. In Western Texas, as elsewhere on the Northern American Continent, the crotalidae, the water moccasin and the copperhead snake, with perhaps the multi-colored king snake included, are the most venomous and dreaded snakes. The balance of a vast variety of snake species are harmless (except they may "scare the wits out of you," when unfamiliar with such, or superstitious), and a large majority are even very useful and elegant looking creatures, as they feed on and destroy all sorts of vermin. Our common water or fish snakes are often confounded with the dangerous type—the black water moccasin. The genuine moccasin is a rather small snake, not much larger than two or three feet, with dark, lead-colored scales, small, piercing eyes, black-yellow stripes on abdominal side, and a very broad, heart-shaped head, which is supplied with long, sharp-pointed and slightly curved poison fangs. I have occasionally, during hunting trips, encountered such. They are ovi-viviparous and, like their viturpurous companion—the rattlesnake—they warn their young offspring during pending danger, when all the young (up to twenty-eight have been counted!) rapidly crawl into the mouth and esophagus of the old snake.

In the annual report of the Smithsonian Institution, United States National Museum (1900), Prof. E. D. Cope reports this on the moccasin snake, or "cotton mouth" (*aucistradon*): "Three species of this genera are known. They are snakes of robust habit, and their bite is highly dangerous. One is terrestrial in habit and the other semi-aquatic. The moccasin is a well-known inhabitant of the Austroriparian region, having a range about equivalent to that of the *Siren lacertina,* and thus characterizing the region. It extends from Southeast Virginia to the Rio Grande and throughout Florida. It ascends the Mississippi to middle Illinois and the Ohio to the Wabash river. It inhabits swamps and the borders of water courses, and catches fishes with ease. It generally seeks the water on being disturbed, but will turn on the pursuer if cornered. Its bite is very dangerous. When about to strike it displays the white interior of its mouth for a short time; hence the name "cotton mouth."

